# Application of Image Analysis to Identify Quartz Grains in Heavy Aggregates Susceptible to ASR in Radiation Shielding Concrete

**DOI:** 10.3390/ma9040224

**Published:** 2016-03-25

**Authors:** Daria Jóźwiak-Niedźwiedzka, Roman Jaskulski, Michał A. Glinicki

**Affiliations:** Institute of Fundamental Technological Research, Polish Academy of Sciences, 5B Pawińskiego, Warsaw 02-106, Poland; rjask@ippt.pan.pl (R.J.); mglinic@ippt.pan.pl (M.A.G.)

**Keywords:** alkali-silica reaction, grain size, heavyweight aggregate, image analysis, radiation shielding concrete, reactive aggregate, quartz

## Abstract

Alkali-silica reaction (ASR) is considered as a potential aging-related degradation phenomenon that might impair the durability of concrete in nuclear containments. The objective of this paper is the application of digital analysis of microscopic images to identify the content and size of quartz grains in heavy mineral aggregates. The range of investigation covered magnetite and hematite aggregates, known as good absorbers of gamma radiation. Image acquisition was performed using thin sections observed in transmitted cross-polarized light with *λ* plate. Image processing, consisting of identification of ferrum oxide and epoxy resin, and the subsequent application of a set of filtering operations resulted in an adequate image reduction allowing the grain size analysis. Quartz grains were classified according to their mean diameter so as to identify the reactive range. Accelerated mortar bar tests were performed to evaluate the ASR potential of the aggregates. The SiO_2_ content in the heavyweight aggregates determined using the image analysis of thin sections was similar to XRF test result. The content of reactive quartz hematite was 2.7%, suggesting that it would be prone to ASR. The expansion test, according to ASTM C1260, confirmed the prediction obtained using the digital image analysis.

## 1. Introduction

Nuclear safety-related concrete structures are composed of several constituents that provide for multiple functions; *i.e.*, load-carrying capacity, radiation shielding, and leak tightness. A comprehensive evaluation of potential aging-related degradation mechanisms that might impair the concrete containment durability revealed the significance of the alkali-silica reaction [1,2,3]. Though this degradation mechanism is well documented for bridges and pavements in particular [4,5,6], its potential consequences on the structural integrity of the containment need to be assessed. This is crucial for potential license renewal beyond the original nuclear plant licensing term. The risk of alkali-silica reaction should be evaluated for new plants at the mix design stage so as to minimize the consequences by the proper material selection. Current generation Portland cements have increased alkali contents [7] that may result in higher content of alkalis in pore water and have a stronger influence on the reactivity of aggregates that were not reactive in the past, and in many countries the availability of good-quality aggregate materials is becoming limited.

Alkali-silica reaction (ASR) is commonly understood as chemical reaction in concrete between hydroxyl ions (OH^−^) of sodium and potassium present in pore solution and certain siliceous rocks and minerals present in some aggregates [8,9]. The development of the alkali-silica gel reaction product can, under certain circumstances, lead to significant swelling and cracking of concrete. It is well established that limiting the availability of reactive silica minerals present in some aggregates and the alkalis coming from Portland cement is an effective means of preventing damage due to ASR. Thus, selecting “non-reactive” aggregates has become one of the major goals during mix design for durable concrete.

Different approaches, including petrographic study, chemical tests, dilatometric evaluation, and accelerated and long-term laboratory tests are used to evaluate the ASR potential of aggregates [10,11,12,13,14]. Since the early work by Stanton [15], numerous investigations have been conducted in order to design a quick and reliable method to establish the susceptibility of some rocks to developing alkali-aggregate reactions [16]. During the work of the RILEM Technical Committee 219-ACS (Alkali Aggregate Reaction in concrete structures: performance testing and appraisal), it has been stated that sedimentary and deformed metamorphic rocks are those that more commonly, but not exclusively, exhibit signs of ASR when used in concrete [12]. Different types of rocks, implying different mineral compositions, microstructural textures, and provenances, exhibit reactive components that are characteristic of each type or group of analysed aggregates were presented in earlier publication [17]. The most reactive forms of reactive minerals in aggregates are strained quartz, amorphous silica, cryptocrystalline quartz, chalcedony, and chert. They may cause deterioration of concrete when the reactive components are present in amounts as small as 1%. In general, aggregates containing crystalline silica are stable, and those with amorphous or very fine-grained silica are reactive. Less reactive forms of silica can eventually cause concrete deterioration after 15 or even 25 years after construction. So, the primary factors influencing alkali-silica reactions include the aggregate reactivity, namely the amount and grain size of reactive SiO_2_ [18] 

The common features of each rock type used in concrete were explained in the context of ASR in [12] and [17]. However, the list of the main types of aggregates didn’t contain heavy aggregate or main minerals present in aggregates that can be used for radiation-shielding concrete. These include aggregates that contain or consist predominately of materials such as barite, magnetite, hematite, ilmenite, and serpentine [19]. Since nuclear shielding structures are classified as high-risk structures [8,20], and the time scale involved reaches 100 years of service (including the plant decommissioning period) there is a need for more advanced tools for ASR risk evaluation, which was just started in [21].

Recent observations of premature deterioration of nuclear shielding concrete structures in Seabrook Power Plant (US, NH), including electrical tunnel, containment enclosure building, control building, and tank farm area have suggested the development of the ASR [22]. The performed petrographic examinations confirmed that ASR was the main reason for concrete degradation [23]. Also, the expansion produced by ASR has been observed in the turbine generator foundation of unit 1, Ikata nuclear power station, Japan [24]. Previous ASR studies on radiation shielding concrete [25] showed that crystalline quartz (or *α*-quartz with a specific gravity of about 2.65), due to irradiation, is converted to distorted amorphous quartz with a specific gravity of 2.27. The decrease of the resistance to nuclear radiation with increasing the SiO_2_ content in aggregates strongly indicates that the deterioration is due to the acceleration of alkali-silica reaction of aggregates in concrete by nuclear radiation [26].

The objective of this investigation is to develop a computer-aided microscopic method of identification of the mineralogical composition of high-density mineral aggregates on thin sections. It is assumed that an application of digital image analysis of minerals on thin sections can largely contribute to improved recognition of reactive components of such aggregates. Thin section analysis is recommended as a routine part of the petrographic examination of aggregates [12,27], usually performed as the essential first stage in the classification of the alkali-reactivity potential of concrete aggregates. The use of image analysis is currently limited to rock types with grain sizes resoluble under the optical microscope and to quartz grains without a high degree of strain [11]. The advantage of image analysis in comparison to the traditional point-counting method can be the speed. The point-counting method proposed by Wigum [28] would take several hours to assess ~200 quartz grains. With the image analysis method used in [11], the assessment of up to 27,606 quartz grains was possible during 10 to 120 minutes. It was found that petrographic image analysis allows the assessment of a higher number of quartz grains in a shorter time without compromising the precision of the results.

## 2. Experimental

### 2.1. Materials

**Figure 1:**
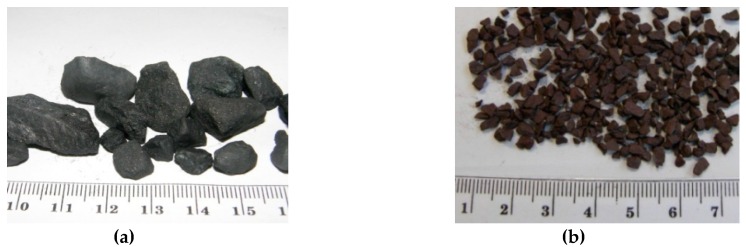
The shape and size of crushed aggregates (**a**) magnetite from Sweden; and (**b**) hematite from Morocco.

Two types of high-density aggregates were tested: magnetite and hematite. Magnetite was collected from Sweden (Minelco AB, Kiruna), and hematite from Morocco (Materials and Techniques SARL, Casablanca). In Figure 1, a general view of tested aggregates is presented. Table 1 presents the mineral composition of aggregates. The Fe_2_O_3_ content in magnetite and hematite was above 85%. The aggregates were characterized with the following densities: magnetite 4.8 g/cm^3^, and hematite 5.1 g/cm^3^. Such density makes them useful for heavyweight concrete, allowing for a designed high density of concretes, usually specified in radiation shielding elements. 

In order to investigate the effect of alkali content in cement on the expansions due to ASR, three levels of total and soluble alkali content in ordinary Portland cement were investigated (Table 2). The content of alkalis in cement did not significantly affect the expansion of the mortar made with highly-reactive aggregate, but apparently had a significant effect on the expansion of the mortar made with the aggregate treated as non-reactive [21]. For further microscopic analysis, the specimens made with cement C1 (the highest content of Na_2_O_eq_) were investigated.

### 2.2. Methods

#### 2.2.1. Thin Sections and Image Acquisition 

The epoxy-impregnated thin sections were prepared from both aggregate and mortars (post-mortem expansion-test specimens). Epoxy resin used for impregnation contained a fluorescent yellow dye. Depending on the aggregate size, small grains as well as post-mortem specimens were first impregnated and then cut into slices, while bigger grains were cut straightaway and then vacuum impregnated. The aggregate and mortar specimens were ground and polished to 20 ± 2 µm thickness thin sections. Two thin sections were cut off from each post-mortem expansion-test specimen. The Pelcon automatic thin section machine was used, and the manufacturing procedure of thin sections was previously described in [29]. The section thickness was verified using the reference patterns for quartz colour in cross-polarized light.

**Figure 2:**
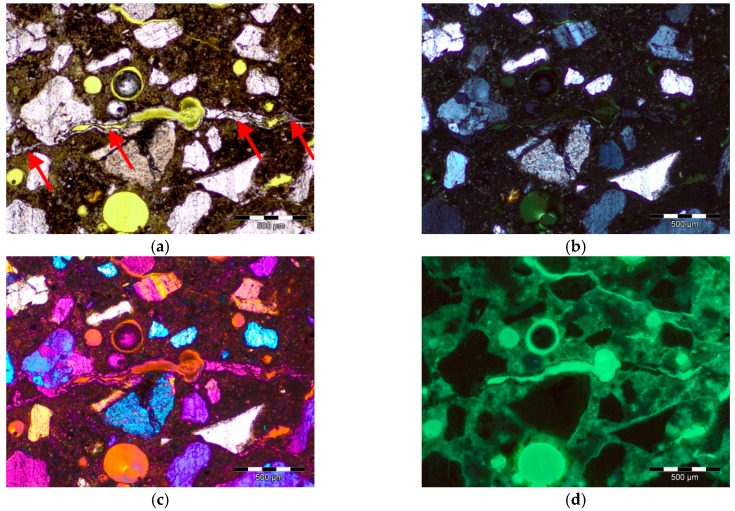
Examples of acquired images of thin section (alkali-silica gel in the matrix) in: (**a**) plane-polarized light (PPL); (**b**) cross-polarized light (XPL); (**c**) cross-polarized light (XPL) with *λ* plate; (**d**) ultraviolet light (UV); the scale bar = 500 µm.

Thin section image acquisition was performed using an Olympus BX51 microscope in plane-polarized light (PPL), cross-polarized light (XPL), XPL with *λ* plate (as applicable), and in ultraviolet light (UV), Figure 2. The *λ* plate is a first-order red plate which consists of a quartz or gypsum plate that is cut parallel to the optic axis, about 62 μm thick, which shows a first-order red interference colour in diagonal position. Due to the high content of ferrum, which is opaque in transmitted light, the identification and analysis of minerals was performed in XPL with *λ* plate mostly, and the information about microstructure of aggregates or their eventual defects like microcracking or open porosity was collected in UV light. Photomicrographs were acquired using an Olympus DP25 digital color camera (Olympus, Tokyo, Japan), automatic moving table Prior ES11BX/B, (Prior Scientific Instruments Ltd., Cambridge, UK, and analySIS software (5.0, Olympus Soft Imaging Solutions GmbH, Munster, Germany).

#### 2.2.2. Accelerated Expansion of Mortar-Bar 

ASTM C1260 Standard Test Method was used for the detection of the potential for deleterious alkali-silica reaction of aggregate in mortar bar exposed to NaOH solution at 80 °C. Three mortar bar specimens were prepared for each aggregate type. The mortar specimens were kept in 23 ± 2 °C and RH ≥ 95% for 24 h. After 24 h in the mould, the mortar specimens were stored for the next 24 h in water at 80 ± 1 °C. After that, their initial length (zero reading) was recorded by a digital extensometer before immersing in 1M NaOH at 80 °C for 14 days. Subsequent specimen length measurements were taken at least three times during the test. The final assessment of the potential reactivity of tested aggregates was carried out on the basis of the relative length increase (the expansion) of mortar specimens, Table 3.

## 3. Image Analysis Procedure

**Figure 3:**
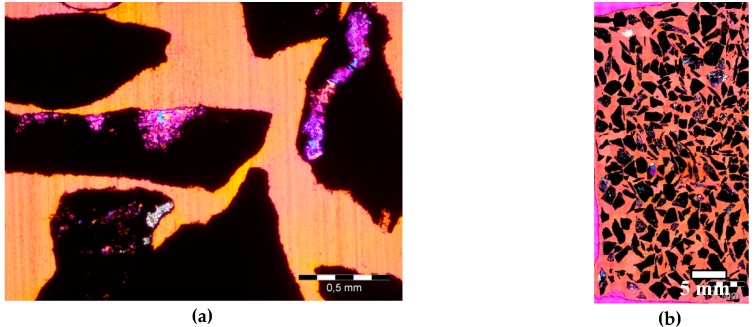
Images of hematite aggregate grains on thin section in XPL with *λ* plate; (**a**) single acquired image, scale bar = 0.5 mm; (**b**) assembled image; scale bar = 5 mm.

The analyzed heavy aggregates, magnetite and hematite, consist mostly of ferrum oxides, which are opaque on thin section in transmitted light, so the identification and analysis of minerals was performed in XPL with gypsum plate. A digital image of a whole thin section is shown in Figure 3, which illustrates a multicomponent image consisting of aggregate grains and surrounding epoxy resin. The separate images (1.5 mm × 2 mm) were automatically acquired and assembled into one image (20 mm × 40 mm), which was then subjected to a process of further analysis to estimate the total content of quartz grains.

To assess the total content of the SiO_2_ crystals in aggregates, it was necessary to distinguish different “phases” (this term is used in the image analysis software) in the assembled image. They correspond to the particular minerals in the aggregate specimen and the surrounding epoxy resin. The most efficient method in the case of ferrous aggregate containing grains of quartz is to define three “phases” on the image: the ferrum oxides, the epoxy resin, and the quartz. The objective is to subtract the area of the first two phases from the whole image area to calculate the total content of the quartz. The consecutive detailed grain size analysis is then performed only on the quartz phase. This is guaranteed by the proper selection of the ROIs (regions of interest) on the images containing only the quartz grains and perchance a thin margin of surrounding ferrum oxide phase which does not affect the results.

**Figure 4:**
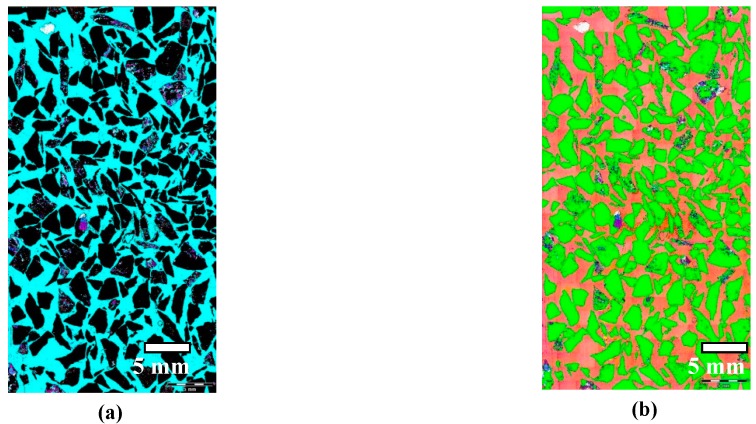
Image of hematite aggregate grains on thin section observed in XPL with *λ* plate; (**a**) after epoxy resin separation-light blue color; (**b**) after ferrum oxides separation-light green color; scale bar = 5 mm.

The procedure of defining the ferrum oxide and the epoxy resin phases in the analySIS software comes down to pointing the selected characteristic small area of the image with the cursor. Then the values of the Red, Green, and Blue components are attributed to the pointed area. The attributed RGB values can be further corrected by including or excluding another areas of the image to obtain more accurate selection of the phase. For the image presented in Figure 3b, the levels of RGB values were set to *R*: 166–255, *G*: 38–255, *B*: 0–226 for fluorescent resin selection, and to *R*: 0–76, *G*: 0–21, *B*: 0–39 for ferrum oxides selection. For better control of the selection process, different colors were attributed to each phase so that it is possible to instantly see the results of the process and correct it. The selection results are presented in Figure 4. After selection of ferrum oxide and epoxy resin phases, analySIS software calculates the area and the percentage of them which subtracted from the whole area of the image gives the total content of quartz.

The distribution of quartz grain size was evaluated using the size categories. It is known that aggregates containing quartz with a small grain size will be more prone to develop deleterious ASR due to an increase in available surface, provided that the increased surface is sufficiently accessible for the pore solution [11,12,30]. When the petrographic texture of a rock is studied, the term microcrystalline is applied to a texture so fine-grained that a petrographic microscope is needed to resolve individual crystals (4–62 μm). The term cryptocrystalline is applied when the texture is too small to be resolvable under petrographic microscope (<4 μm). The threshold applied to rocks with micro and cryptocrystalline quartz texture is also different in each country [31]. So, the classes of the quartz size were established according to the Norwegian Criteria, as shown in the research of Alaejos and Lanza [32]. The reactive forms of quartz have been classified depending on the crystal size as innocuous quartz: >130 μm; doubtful quartz: 60–130 μm; reactive quartz: 10–60 μm and highly reactive quartz: <10 μm.

**Figure 5:**
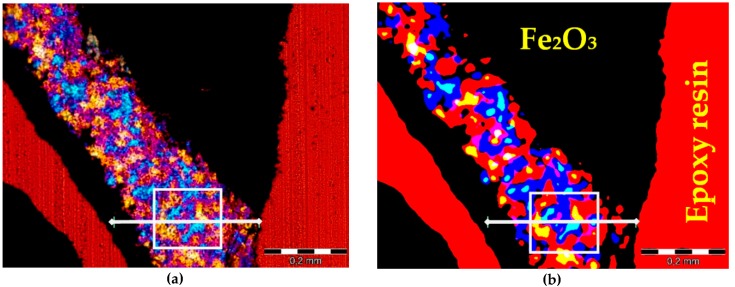
Representative image of hematite aggregate grain on thin section observed in XPL with *λ* plate; (**a**) original image; (**b**) image after filtering procedures; scale bar = 200 µm.

To identify and assign quartz grains to proper classes, the high quality separated images of size 1.5 mm × 2.0 mm were used. Three filter operations of analySIS software were used to prepare the pictures for image analysis. They were in sequence: Remove Noise filter, Average filter, and Posterize filter. The first was used (with parameter value 32 from parameter range 1–128) to remove very small aggregations of pixels inside a single quartz grain area that could be falsely recognized as a separate grain of a very small size. The second filter was used (with parameter value 32 from parameter range 1–128) to decrease a variability of colors within each grain of quartz. And finally the third filter was used (with parameter value 2 from parameter range 1–64) to assign a single color (the closest from the color range) to each distinguished quartz grain. The example of the original image and the result of its filtering transformations is presented in Figure 5.

**Figure 6:**
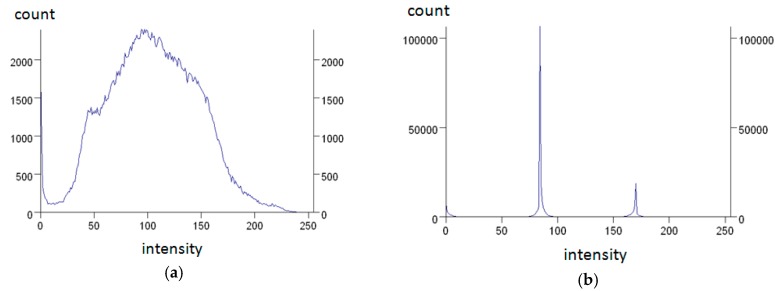
Histograms computed from a selected area of hematite aggregate grains (marked rectangle) on thin section taken from: (**a**) original image; (**b**) image after filtering procedures (see Figure 5).

**Figure 7:**
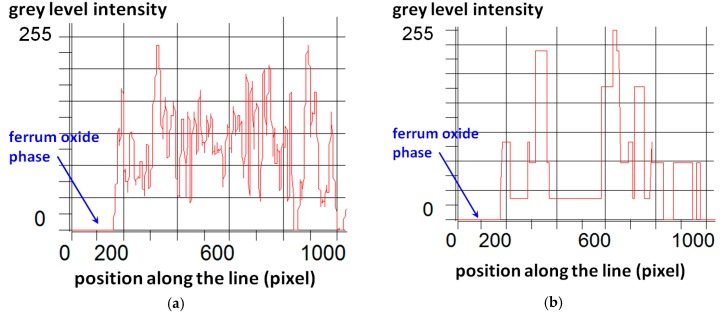
Grey level intensity profile along the selected horizontal line taken from: (**a**) original image; (**b**) image after filtering procedures (see Figure 5).

To illustrate the effects of filtering operations, the images were first converted from true-color images into grey-value images. In Figure 6 the computed histogram of intensity values over the selected area of quartz phase (presented in Figure 5) is shown. The histograms are based on the grey-value image intensity. Vertical axis refers to the number of pixels of the given intensity value. After filtering operations, the grey level variability is reduced to the narrow range around two intensity levels. In the Figure 7, results of the filtering procedures are presented differently, as a grey values profile computed for selected horizontal line marked in Figure 5. This time, the vertical axis refers to the intensity of grey value of pixels along the line. Comparing Figure 7a,b, a significant decrease of the initial diversity of the grey value intensity can be noticed. Flat, horizontal segments of the graph in Figure 7b can be identified as a size of the single grain of quartz measured along the selected line. The segments of intensity value equal to 0 represent the ferrum oxide phase.

Further analysis was performed on the processed images to classify the quartz grains according to their mean diameter. The new set of “phases” was defined in accordance with the colors of quartz grains obtained after the filtering operations. On the basis of the so-distinguished “phases”, the Detect command of the analySIS software was executed. As a result, the number and the equivalent mean diameter of separated quartz grains was obtained so as to allow grain size classification.

## 4. Test Results and Discussion

### 4.1. Aggregate

The content of the quartz in magnetite and hematite aggregates was estimated using thin section analysis. The total content of the quartz and its content at the selected size range as related to alkali-silica reactivity are shown in Table 4. As a SiO_2_ crystal size, the mean grain size has been established because the mean grain size of quartz appears to be the most favourable quantitative parameter related to the expansion of different rock types [28].

The total quartz content in both magnetite and hematite aggregate is similar to the content determined using XRF method on powdered aggregate specimens. The thin section analysis revealed slightly higher SiO_2_ content (3.6%) in magnetite and 11.6% in hematite than the XRF method (~3.2% in magnetite and ~10.7% in hematite). Obviously it is difficult to compare the size of the analyzed aggregate specimens: the area of 20 mm × 40 mm on thin section in 2D and the mass of ~5g of powder in XRF method. The obtained results from thin section and XRF cannot be compared directly, but they provide a similar estimation of the total content of quartz in heavy aggregate.

**Figure 8:**
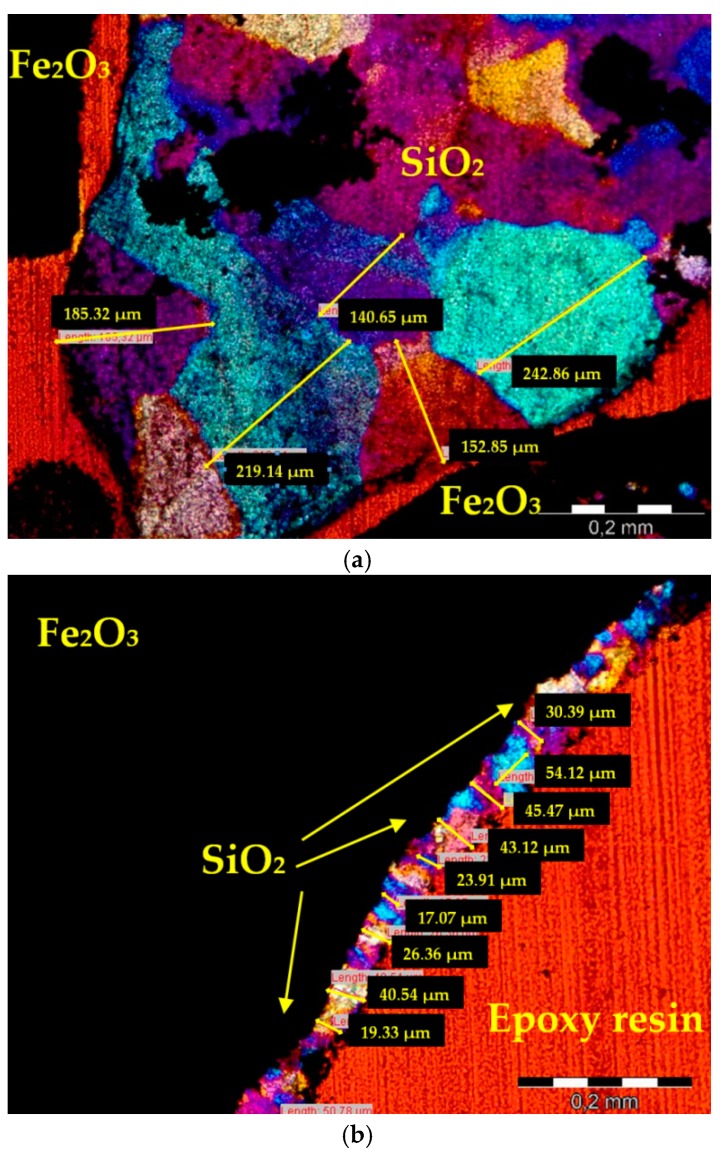
An example of the thin section of hematite specimen in XPL with *λ* plate; (**a**) innocuous quartz: >130 μm; (**b**) reactive quartz: 10–60 μm; scale bar = 0.2 mm.

The total quartz content does not give a good indication of the potential reactivity of heavy aggregate because such data does not provide sufficient information on the amount of potentially reactive silica minerals. The size of the SiO_2_ crystals in heavy aggregates was estimated on thin sections. The example of the hematite thin section in XPL with *λ* plate with two different sizes of visible quartz grains is shown in Figure 8.

The detailed analysis of the sizes of quartz grains revealed that hematite aggregate could be prone to ASR due to the increased content of the reactive quartz in the size range 10–60 μm. The content of this kind of grains in hematite amounted to 2.67%, while in magnetite it was only 0.13%. It is known that the grain size reduction of quartz enhances reactivity by increasing the surface area of quartz grain boundaries available for reaction [28]. Earlier studies done by Alaejos and Lanza [32] showed that when the content of quartz crystals between 10 and 60 μm is taken into account, not only highly reactive quartz <10 μm, the expansion due to ASR is larger. At the same time, quartz crystal size greater than 60 μm (doubtful quartz: 60–130 μm) has no effect on expansion, so it can be considered innocuous. The investigation revealed the individual limit for each reactive component: 8.3 vol.% of quartz 10–60 μm; and 2.6 vol.% of quartz <10 μm (corresponding to 0.1% expansion at 14 days).

There is not enough data to show the correlation between the mean grain size of quartz and the average expansion after 14 days, but the results of this investigation fit in the correlations obtained in [30,31]. The findings confirm earlier assumptions [33] that it is clearly incorrect to consider rock type as a criterion for an aggregate’s potential for reactivity. It is important that attention should be focused on the mineral constituents of the rock itself.

### 4.2. Mortars

**Figure 9:**
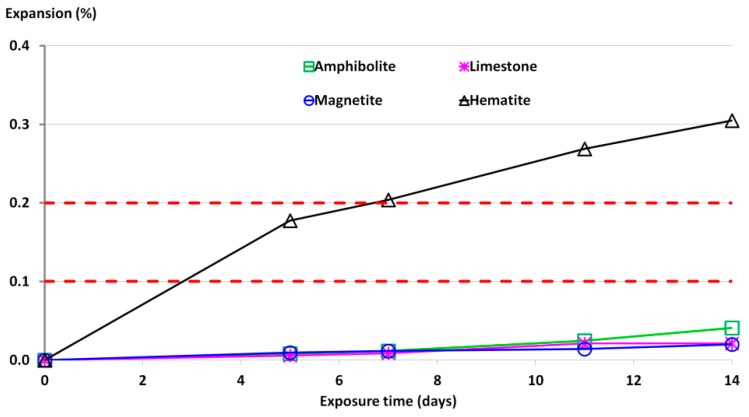
Expansion of mortar bars stored in 1M NaOH at temperature 80 °C during 14 days

The development of expansion for tested aggregates is presented in Figure 9, based on an average from three mortar-bars. As a reference non-reactive aggregate, an amphibolite and limestone aggregate was chosen. Each result is the average of three mortar bar measurements. For all tested specimens the standard deviation was lower than 20%. Dashed red lines indicate the limit for an assessment of reactivity of aggregate per ASTM C1260 (Table 2).

The expansion of all tested aggregates increased with increasing test duration, but the rate of expansion over time varied depending on the aggregate. The results show that the hematite mortar exhibited the highest expansion. The expansion development was faster and more extensive for hematite aggregates than for magnetite aggregate. The hematite mortar bars expansion was extremely fast and high, and after three days maximum, exceeded the 0.1% limit. All the hematite mortar bar specimens after 14 days of standard storage showed an expansion of about 0.3%–0.35%, which classified hematite as very reactive aggregate. The lowest expansion was measured for magnetite mortar bars (0.02%) while the reference aggregate, amphibolite, exhibited the expansion of 0.04%.

In Table 5, the correlation between the content of reactive quartz in heavyweight aggregates and the expansion of mortar bar specimens as per ASTM C1260 is presented. A very small content of highly reactive quartz (0.08%) and reactive quartz (2.67%) in hematite aggregate caused the occurrence of ASR. These are smaller values than in studies done by Alaejos and Lanza [32], but they tested quartzites, quartzarenites, and limestones with chert rocks, and they stated that their results correspond to the tested aggregates and that other different rock types should be tested to confirm these results.

**Figure 10:**
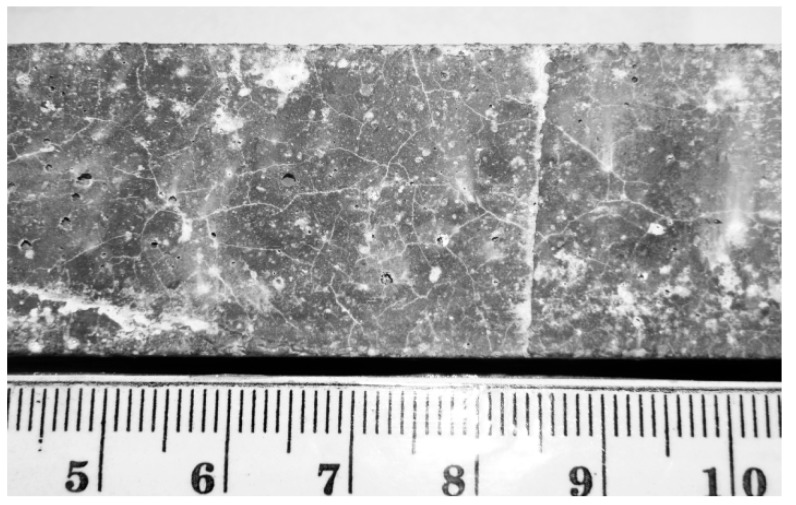
Alkali-silica reaction (ASR) characteristic map of cracking at the surface of hematite specimen after Accelerated Mortar Beam Test (AMBT).

In Figure 10, the characteristic map of cracking due to alkali-silica reaction expansion at the lateral surface of a hematite mortar bar is shown.

**Figure 11:**
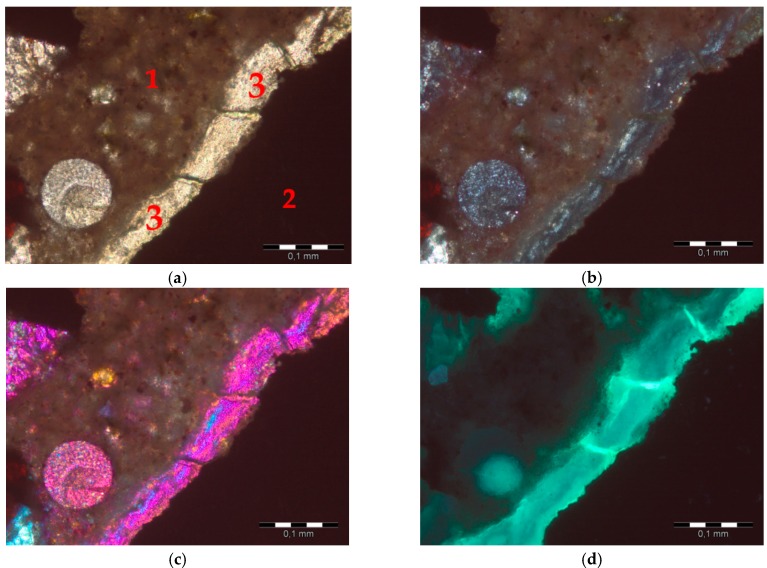
Alkali-silica gel in the matrix in mortars with hematite aggregate: (**a**) plane-polarized light (PPL); (**b**) cross-polarized light (XPL); (**c**) cross-polarized light (XPL) with *λ* plate; (**d**) ultraviolet light (UV); 1-cement matrix, 2-hematite, 3-alkali-silica gel; scale bar = 100 µm.

**Figure 12:**
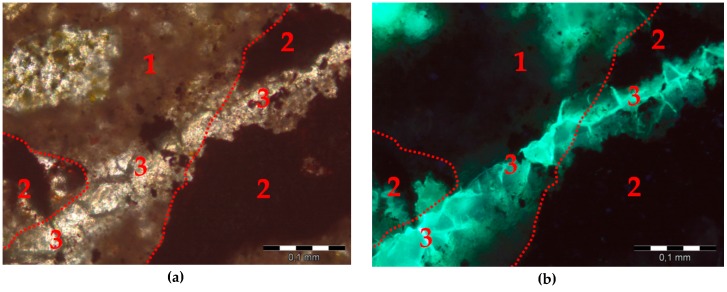
Alkali-silica gel in the matrix in mortars with hematite aggregate with a marked dotted line probable original contour hematite aggregate: (**a**) plane-polarized light (PPL); (**b**) ultraviolet light (UV); 1-cement matrix, 2-hematite, 3-alkali-silica gel, scale bar = 100 µm.

After the accelerated expansion test, mortar bars were selected for thin sectioning, and the post-mortem analysis on thin sections was conducted. The evidence of alkali-silica reaction (cracking and microcracking) was observed in the specimens containing hematite aggregate only, as shown in Figure 11. The characteristic cracked alkali-silica gel was present in small air-voids, in the matrix where it formed a link between reactive grains (Figure 12). More detailed analysis of the post-mortem specimens will be performed using the Scanning Electron Microscopy/energy dispersive X-ray spectroscopy (SEM–EDS) technique to identify the alkali-silica gel composition.

No presence of ASR gel was revealed in the specimens containing other types of aggregates; matrix cracking was not detected either. The accelerated mortar bar expansion data was found coherent with thin section observations, revealing the presence of ASR gel and the increased content of the reactive quartz of the size range from 10 to 60 μm in tested aggregates. One should consider the limitations of the observed correlation of test results—it is limited to a case of minerals mainly composed of two phases, one of them being opaque in transmitted light. The developed method of testing and image analysis was found effective particularly for minerals rich in ferrous phases. However, the obtained distribution of quartz grain size should be understood as related to an effective size; *i.e.*, corresponding properly to accelerated expansion of mortars. The accessibility of the alkaline pore solution to reactive silica is the crux of the issue of aggregate reactivity [9]. If the reactive silica is contained as fine particles distributed within a non-reactive mineral phase, the reaction might occur slowly. Apart from the increased content of the reactive quartz within the range 10–60 μm, its distribution from the grain boundary can play an important role, as well as the presence of microcracks in the aggregates. The use of digital image analysis can also be beneficial to evaluate such features.

## 5. Conclusions 

The following conclusions can be drawn.

1. An experimental method of quantitative evaluation of the content of quartz in heavyweight aggregates and its grain size distribution was developed. The method, based on microscopic image analysis of thin sections, studied in cross polarized light with *λ* plate, was effective in quartz characterization. Image processing consisting of identification of ferrum oxide and epoxy resin, and subsequent application of a set of filtering operations, resulted in an adequate image reduction allowing the grain size analysis.
2. The content of quartz in polymorphic hematite and magnetite aggregates, evaluated using the image analysis method, was quite close to results of XRF analysis on powder specimens, the absolute difference being 0.4% and 0.9% for magnetite and hematite aggregates, respectively.
3. The presence of the reactive quartz of the size range from 10 to 60 μm was revealed in tested heavy aggregates. The content of the reactive quartz grains was 2.67% and 0.13% for hematite and magnetite aggregates, respectively.
4. Accelerated mortar bar tests revealed that hematite aggregates were prone to ASR, since the observed expansion of mortar specimens reached 0.30%–0.35% at 14 days of exposure to NaOH. For a higher content of reactive quartz grains, an increased expansion of mortar specimens was observed.
5. The post-mortem analysis of thin sections prepared from mortars after ASTM C1260 test confirmed the presence of ASR gel, both in the matrix and in hematite aggregates.

An elaborated image analysis method on thin sections is considered useful to support the selection of heavyweight aggregates for the long-term performance of concrete in nuclear shielding structures.

## Figures and Tables

**Table 1:** The main constituents of magnetite and hematite aggregate after XRF method applying the X-ray fluorescence (XRF) method.

Main Mineral Constituents, %	Magnetite Aggregate		Hematite Aggregate	
	Supplier Data	Test Results	Supplier Data	Test Results
SiO_2_	3.00	3.39	11.50	9.83
Fe_2_O_3_	90.80	93.72	85.08	86.74
Al_2_O_3_	0.40	0.51	0.90	0.68
CaO	2.50	1.72	0.05	0.02
K_2_O	0.20	0.10	0.40	0.27
Na_2_O	0.30	0.19	0.02	0.03

**Table 2:** The main chemical constituents in tested cements, in wt.%, by XRF [21].

Main Chemical Constituents	Cement 1 C1	Cement 2 C2	Cement 3 C3
SiO_2_	22.20	19.03	21.48
Al_2_O_3_	5.30	4.84	4.80
Fe_2_O_3_	3.00	3.22	2.62
CaO	66.30	63.64	65.60
MgO	1.30	1.15	0.87
SO_3_	0.60	2.97	2.84
Na_2_O	0.24	0.21	0.12
K_2_O	0.82	0.53	0.47
Total Alkalis, Na_2_O_eq_	0.78	0.56	0.43
Water-Soluble Alkalis	0.54	0.48	0.37
Loss of ignition (LOI)	3.5	3.34	1.12

**Table 3:** Assessment of Reactivity of Aggregate per ASTM C 1260 [30].

Expansion, %	Aggregate Reactivity
<0.1	Innocuous
0.1–0.2	Inconclusive
>0.2	Potentially deleterious

**Table 4:** The total quartz content and the content of the reactive forms of quartz in tested heavy aggregates classified depending on their crystal size by image analysis.

Cristal Sizes	Magnetite Aggregate (%)	Hematite Aggregate (%)
total quartz grains	3.60	11.62
innocuous quartz: >130 μm	3.24	7.41
doubtful quartz: 60–130 μm	0.22	1.46
reactive quartz: 10–60 μm	0.13	2.67
highly reactive quartz: <10 μm	0.01	0.08

**Table 5:** The correlation between the content of reactive quartz in heavyweight aggregates and the expansion of mortar bar specimens as per ASTM C1260.

Heavyweight Aggregate	Highly Reactive Quartz <10 μm, %	Reactive Quartz 10–60 μm, %	Mortar bar Expansion after 14 days, %
Magnetite	0.01	0.13	0.02
Hematite	0.08	2.67	0.31
